# Pediatric High Labial Frenum Management: Utilizing a Diode Laser for the Transformation of the Smile of an Eight-Year-Old Child

**DOI:** 10.7759/cureus.63346

**Published:** 2024-06-28

**Authors:** Rashi S Patil, Ishani Rahate, Punit Fulzele, Rashi Dubey, Ramakrishna Yeluri, Dhruvi Solanki

**Affiliations:** 1 Department of Pediatric and Preventive Dentistry, Sharad Pawar Dental College and Hospital, Datta Meghe Institute of Higher Education and Research, Wardha, IND

**Keywords:** diode laser, pediatric patients, spacing, frenectomy, midline diastema

## Abstract

Median diastema is a physiological occurrence that is frequently seen in the maxillary jaw. Therefore, a median diastema has been associated with a wide range of etiological variables, including thumb sucking, supernumerary teeth, tongue thrusting, some dentoalveolar discrepancy, and hypodontia. Patient esthetic and function demands are both negated by the labial frenum’s abnormal location in relation to the maxillary anterior teeth, which results in diastema and gingival recession. Lasers are now being used in several fields of dentistry as an alternative to conventional scalpel operations. Frenectomy can be done through the use of electrosurgery, laser surgery, or the classic scalpel technique. This pathological frenum can be very well excised with a diode laser. Due to its applicability, sufficient coagulation, lack of suture requirements, and reduced discomfort and inflammation, the diode laser can be utilized in pediatric dentistry. High-connected midline diastema has remained a subject of debate when it comes to management and the right time to intervene and treat it. Both orthodontists and pediatric dentists agree that frenectomy should not be done after the closure of the orthodontist gap or before the appearance of the permanent canine teeth. However, several conditions, including the child’s psychological status, parents’ concerns, the closure’s unpredictable effects on the future, and the expense of combined therapies, may lead to an early intervention for therapy during the primary or mixed dentition. In this specific scenario, a child who was eight years old underwent a diode laser frenectomy. After seven days, a follow-up examination showed normal position and attachment of the frenum and no signs of infection at the site of surgery.

## Introduction

The labial area of the frenum is a fibrous mucosal tissue fold that connects the periosteum of the maxillary and mandibular bones, gingiva or alveolar mucosa, and cheek and lip regions [1]. To avoid excessive pull at the marginal gingiva, there is normally a proper frenal attachment at the terminal part of the mucogingival junction [2]. Sewerin divided the frenum attachment into a) normal frenum; b) normal frenum with nodules; c) normal frenum with an appendix; d) normal frenum with nichum; e) bifid labial frenum; f) persistent tectolabial frenum; g) double frenum; and h) wedger frenum [3]. Midline diastema, gingival recession, loss of interdental bone, poor lip movement, inability to brush well, and misaligned teeth are complications resulting from improper frenum attachment [4]. A frenectomy, or frenotomy, is the available treatment for aberrant frenal attachment. A frenectomy involves removing the frenulum completely and restoring its connection to the underlying bone. A frenotomy consists of making an incision and realigning the frenal attachment [5]. The frenum attachment of newborns is near the alveolar ridge or the papillae at birth [6]. Despite this, several reasons often lead to the removal of a child’s maxillary frenum: oral incompetence that makes the child unable to make bilabial speech sounds (/b/, /p/, /m/, /w/), spoon handle feeding, habitual open mouth breathing, full lips, and diastema. The maxillary frenum of some newborns may limit their ability to breastfeed or take a bottle [6]. There are seven frenula usually found in the oral cavity: four buccal (cheek) frenula, the mandibular labial frenulum, the lingual frenulum, and the maxillary labial frenulum. They are meant to anchor the tongue and support the lower and upper lips. There is a small flap of tissue known as the lingual frenum, which runs from the floor of the mouth to the undersurface of the tongue. At the base of the frenum is a ‘V'-shaped flap on the floor of the mouth, which embraces several salivary gland ducts [6].

In dentistry, it is apparent that there is precedence for the conservative treatment of oral disorders with laser technology. The diode apparatus is still categorized as the new laser technology for application in the field of dentistry. Diode lasers of wavelength 980 nm, for instance, have the following benefits: they are helpful in the control of bleeding, pain, and inflammation [7]. The choice of material for the fabrication of these devices is through the use of solid-state materials such as gallium arsenide, aluminum, and indium. The diode laser is one type of laser that is used to change electrical energy into light energy [8]. The creation of laser light involves the accumulation of the precursors for stimulated photon emission and the subsequent amplification of these photons through an optical gain cascade mechanism. Lasers can produce extremely narrow beams of heat energy that do not diffuse much as the distance from the source of energy is increased since they have the characteristics of high power density as compared to normal light sources [9]. The laser beam to the cells leads to processes such as warming, welding, coagulation, protein denaturation, drying, vaporization, and carbonization [10]. Lasers can be used in oral surgery treatments such as frenectomy, excision of gingival hyperplasia of impacted or partially erupted tissue, photodynamic therapy for malignancies, photostimulation for herpetic lesions, vestibuloplasties, excision of soft tissue tumors, and excision of hematomas [11, 12]. Moreover, lasers are applied in hard tissue surgery such as endodontic root canal decontamination, caries prevention, restorative material removal and solidification, cavity preparation, dental sensitivity management, growth regulation, bleaching, and diagnostic processes [13, 12]. In pediatrics, the performance of operations without the use of infiltrating anesthetic agents is new and has become an extremely important procedure. It should also be noted that along with classical treatment methods, diode laser therapy is also effective in treating inflammation in children. There is always some sort of threat or worry regarding a needle or any sharp instrument for any child, but with the innovations in the field of pediatric dentistry, this newer laser technique can help the child overcome their fear [14].

## Case presentation

**Figure 1:**
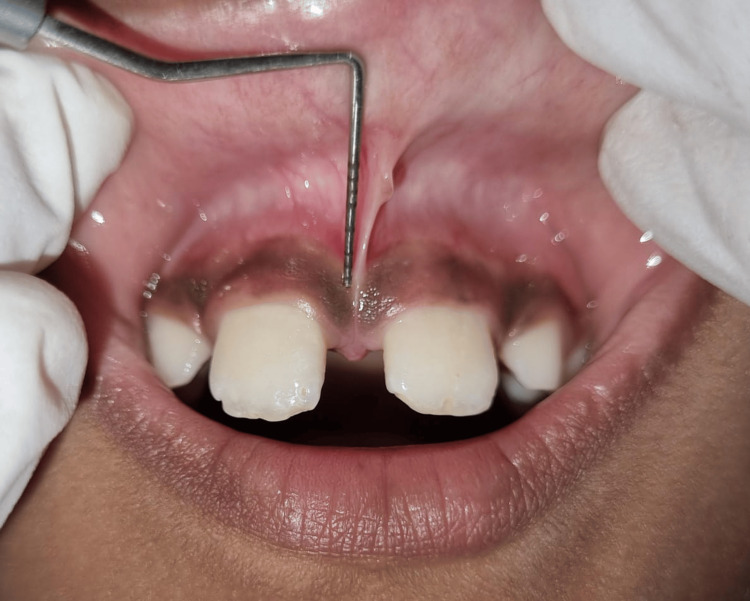
The preoperative photograph shows a high frenum attachment with a mid-line diastema.

An eight-year-old male patient reported to the outpatient Department of Pediatric and Preventive Dentistry with the chief complaint of spacing between the two upper front teeth of the jaw. On extraoral examination, no gross asymmetry was observed, lips were competent, the retrognathic mandible was seen, the facial profile was convex, and there was bilaterally smooth and synchronized temporomandibular joint movement. The patient also did not have any significant medical history or dental history present. Intraoral examination revealed that there was Class II molar relation on both sides, with spacing between teeth 11 and 21, 11 and 53, and similarly with teeth 21 and 63. The patient was in the mixed dentition phase and had a high labial frenum attachment. There was a 5 mm broad midline diastema with a high attachment and also a frenal tissue tag. As per Placek Mirko's classification, this case was identified as a papillary-type frenulum attachment (Figure 1).

**Figure 2:**
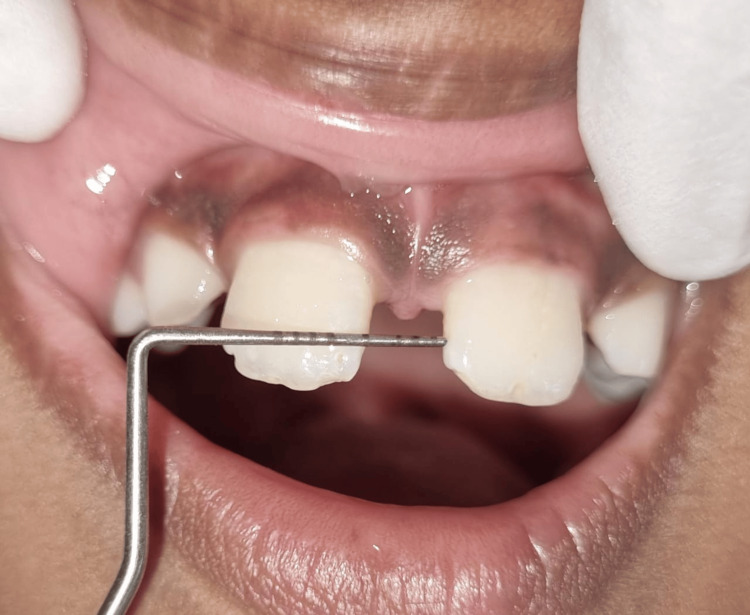
The measurement shows 5 mm midline diastema between teeth 11 and 21.

A 5 mm broad midline diastema with a high attachment of the papillary-type frenum was noted. Furthermore, on both sides, 1-2 mm gaps were seen between the canines and central incisors (Figure 2).

**Figure 3:**
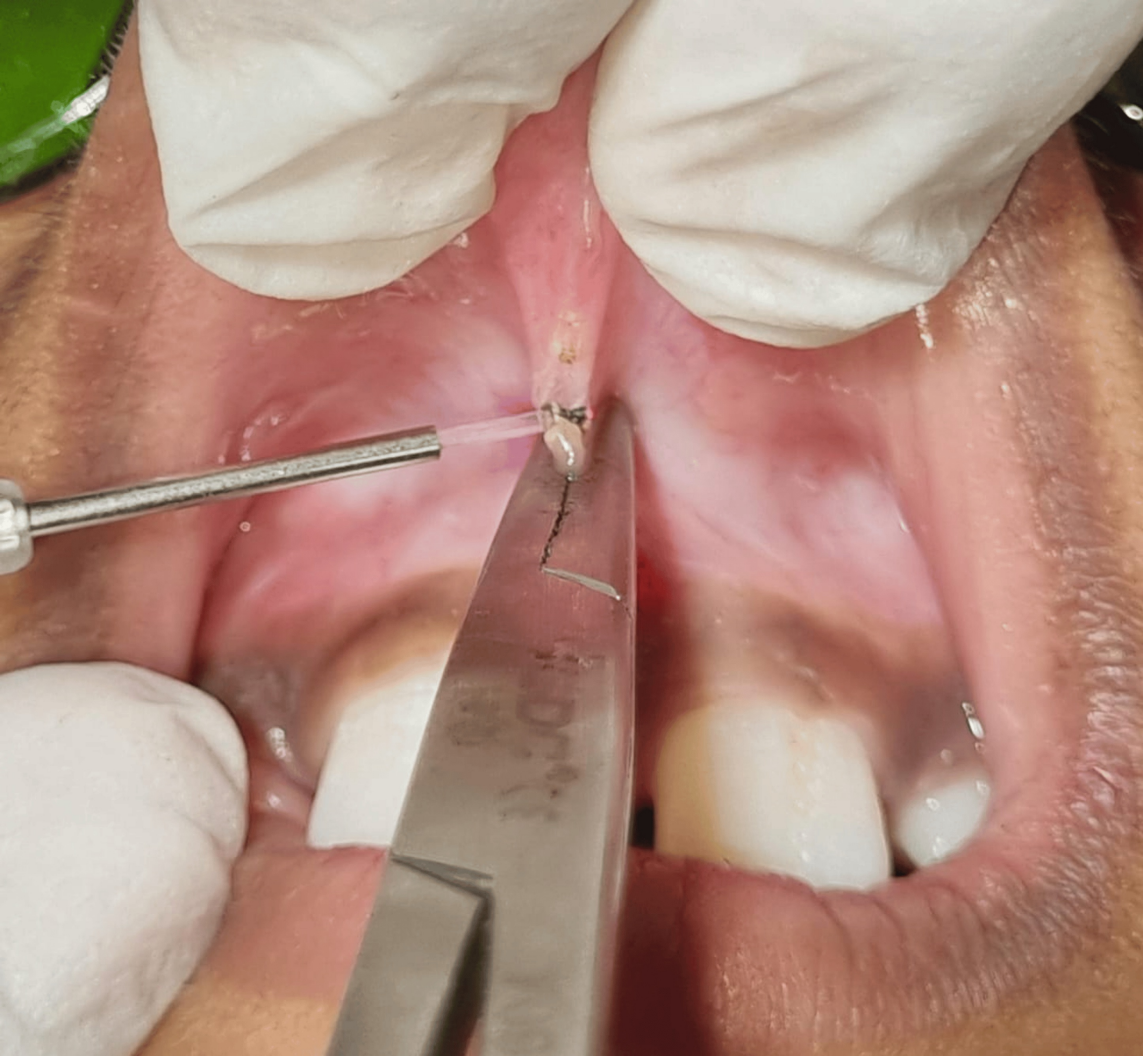
Labial frenum was held using a hemostat, and frenectomy was performed with a diode laser.

Normal color and texture gingival pigmentation was seen. No abnormalities were seen upon inspection of the teeth and other soft tissues. After initial oral prophylaxis, the patient was then referred to the Department of Oral Pathology for a complete blood count (CBC) investigation, and reports were in the normal range. Before undergoing surgery, written consent was obtained from his parents. The patient was then recalled after eight days for a labial frenectomy. Under all aseptic conditions and precautions, the local anesthetic agent (lignocaine with adrenaline 1:100000) was administered after the application of topical lignocaine gel. To protect themselves from the laser radiation, the patient as well as the operator were required to wear goggles before the treatment. Before the procedure, the 400 μm surgical laser tip was employed, and a 980 nm (EPIC X Diode Laser, Biolase, CA) tip was then applied to the bottom of the labial frenum vertically, and the frenectomy was performed (Figure 3).

**Figure 4:**
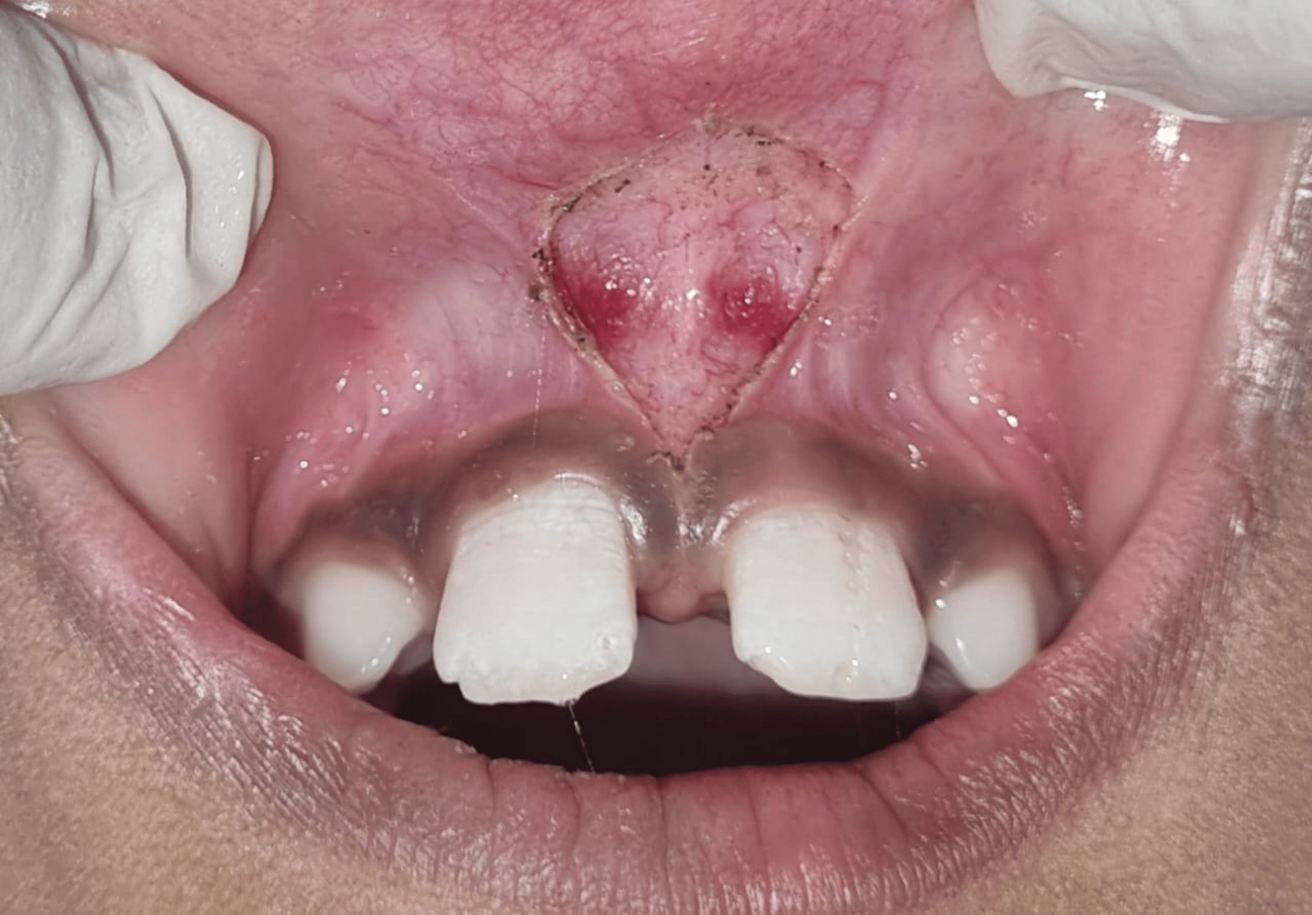
Diamond-shaped wound after surgery

After relieving fibrous attachment from the labial frenum, hemostasis was achieved without suturing. Following the surgery, postoperative instructions as well as analgesics were given to patients (Figure 4).

**Figure 5:**
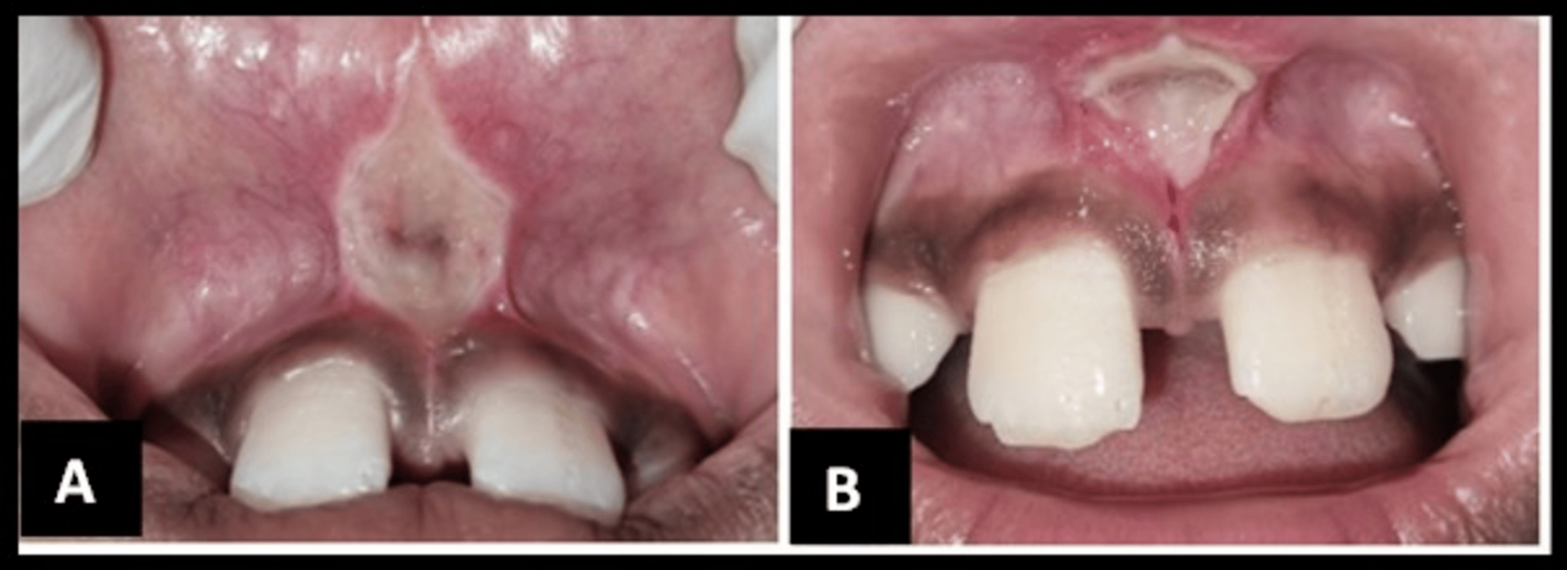
Follow-up (A) after three days and (B) after a week.

A follow-up was done after three days, seven days, and a month during which adequate healing was observed upon examination (Figure 5).

**Figure 6:**
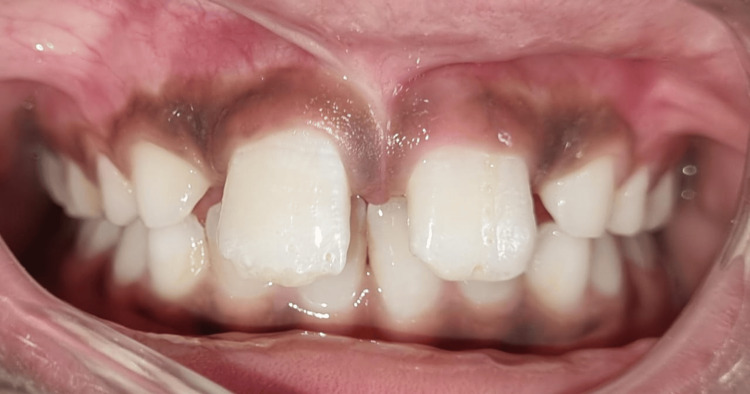
Follow-up photograph taken after one month

Complete healing was seen after one month of surgery(Figure 6).

Currently, the patient is undergoing orthodontic treatment for upper anterior spacing.

## Discussion

The upper labial frenum, or frenulum labii superioris, is the fold of the mucous membrane where the lip connects to the alveolar process [15]. Abnormal frenum attachment can produce recession, loss of papilla, diastema between teeth, difficulty brushing, and malocclusion that may give rise to psychological problems in children [16]. As for the distribution rate of the various types of aberrant frenum attachment, the gingival type has the highest rate (49.5%). The second is mucosal (38%), and papillary (9.8%), while papillary penetrating was the least observed (1.9%) [17]. Some suggested treatment methods in the literature include lasers, bipolar diathermy, and surgical blades, among others. Surgical frenectomy has its risks, which include bacterial infections, bleeding, damage to the neighboring salivary ducts in the tongue region, soreness, swelling, and scarring of the tissue [18]. A surgical frenectomy has risks such as bleeding, infection, damage to other structures, particularly the salivary ducts near the tongue tie, pain, swelling, anaphylactic reaction to anesthesia, and recurrent tying over of the frenum.

The abbreviation ‘laser’ is an acronym for Light Amplification by Stimulated Emission of Radiation and broadly encompasses any apparatus for amplification of light to the creation of a very focused, correlated light wave [19]. Laser radiation can be classified as soft laser radiation or as hard laser radiation. The cold (thermic) energy lengths of soft lasers are thought to activate cellular activity. The majority of these soft lasers are constructed using diodes and, as the manufacturers of these lasers have stated, could be beneficial for healing tissue by decreasing pain, edema, or inflammation. Some examples of soft lasers are the helium-neon laser (He-N) and the gallium-arsenide (Ga-As) laser. Surgeon-usable lasers are ideal for cutting through both the soft and hard tissues of the body. The current models are capable of using a type of flexible fiber-optic cable for the transfer of their power. Argon lasers (Ar), carbon-dioxide lasers (CO2), and neodymium-doped yttrium-6-aluminum garnet (Nd:YAG) are some of the common examples of hard lasers. The most commonly used lasers in dentistry include holmium yttrium aluminum garnet (HO: YAG), neodymium-doped yttrium-6-aluminum garnet (Nd: YAG), carbon-dioxide lasers (CO2), erbium-doped yttrium aluminum garnet (Er: YAG), diodes, and argon lasers [20]. The characteristic of the diode laser is its ability to selectively ablate a thinner part of the upper epithelium and, subsequently, cause a sterile inflammatory reaction. In addition to this, the least invasiveness of the procedure is observed to the extent that the bone and periosteum under the gingiva that is being treated are slightly affected. Some benefits of laser postoperative management include reduced discomfort, no blood loss, the development of scar tissues, and enhanced tissue healing, which were noted in laser-assisted frenectomy [21].

In the current case, we have conducted a laser-assisted frenectomy rather than a conventional surgical one, taking into consideration the child's age and reducing postoperative conditions. Parents are concerned about the child's aesthetic in the ugly duckling stage. There was a need to counsel them regarding the mixed-dentition phase. The patient has an end-on-molar relationship on both sides, so orthodontic consultations were made. Currently, the patient is undergoing orthodontic therapy. The patient is kept on hold and recalled for further treatment.

## Conclusions

Early childhood frenum excision performed with a laser minimizes surgical time, eliminates the need for sutures, causes no pain or discomfort after surgery, leaves no scars, and does not recur. However, it remains a dependable substitute because it is a satisfactory, safe, and effective option for soft tissue surgeries like frenectomy. It's a better option for minor surgical procedures in children.
